# Relationship Between Cholesterolosis and Pancreatitis

**DOI:** 10.1155/1991/51089

**Published:** 1991

**Authors:** John P. Neoptolemos, Brian Isgar

**Affiliations:** 1 Reader in Surgery University of Birmingham Consultant Surgeon, Dudley Road Hospital UK; 2 Research Registrar University Department of Surgery Dudley Road Hospital, B18 7QH Birmingham UK


### RELATIONSHIP BETWEEN CHOLESTEROLOSIS AND PANCREATITIS

### ABSTRACT

*Paricio, P.P., Olmo, D.G., Franco, E.P., Gonzalex, A.P., Gonzalex, L.C. and Lopez, J.P. (1990) Gallbladder cholesterolosis: an aetiological factor in acute pancreatitis of uncertain origin. Br. J. Surg; 77: 735–736.*

**To investigate the possible relationship between gallbladder cholesterolosis and acute pancreatitis, we studied 3797 cholecystectomy specimens and found 55 cases of gallbladder cholesterolosis unassociated with biliary lithiasis. From the reviewed case notes, 27 of these patients presented with recurrent attacks of acute pancreatitis which disappeared after cholecystectomy (follow-up 65:1 months). A microscopic study revealed frank cholesterolosis in all cases with a pseudopolyp transformation of the mucosa, some polyps reaching a diameter of 2 mm. We postulate that the mechanism could be temporary impaction of cholesterolosis polyps at the sphincter of Oddi and suggest that patients with recurrent attacks of acute pancreatitis and negative aetiological investigation must be considered as at high risk of having gallbladder cholesterolosis and that they could benefit from cholecystectomy.**

### PAPER DISCUSSION

KEYWORDS: Cholelithiasis; gallstones; pancreatis; acute; cholesterolosis

By far and away, the commonest causes of acute pancreatitis are gallstones and alcohol[1–5]. Although numerous other causes of acute pancreatitis are well recognised, the third largest group is "idiopathic" meaning of unknown aetiology. The relative incidence of idiopathic pancreatitis varies from 9% to 39%[1–7]. In these types of studies the size of the idiopathic group falls inversely in relation to the number of investigations undertaken and in particular to those investigations looking directly at the biliary tract and pancreas. Thus in a study of thirty five patients with acute pancreatitis who had normal ultrasonography and oral cholecystography, ERCP revealed a definite aetiological factor in 12 cases including seven with gallbladder stones[3].

Idiopathic pancreatitis remains a significant clinical problem not only because of its relative high incidence and because nothing can be done knowingly to prevent further attacks, but also because it is a particularly lethal variety[8]. Thus the present paper by P. Parilla Paricio and colleagues is of particular interest.

The histopathological reports on 3797 cholecystectomy specimens obtained in a ten year period up to December 1988 were reviewed. Amongst these were 434 cases of cholesterolosis or 11.4%, a figure which lies at the extreme upper level of previously published work. Gallstones were noted in 379 cases (87%) and none in the remaining 55 cases (13%). Of those with gallstones there were 98 patients (25.8%) who by the authors' definition gave a history of acute pancreatitis. In contrast, 27 patients (49%) with cholesterolosis and no gallstones had a history of pancreatitis. Although no statistical analysis was undertaken by the authors, this difference is significant ($X^2$ = 12.64, df = 1, $p < 0.001$). The aforementioned patients had no further attacks following cholestectomy.

The authors postulate that the mechanism of acute pancreatitis in these patients was due to temporary obstruction of the sphincter of Oddi by cholesterolosis polyps. It was recommended that cholesterosis should be looked for radiologically or ultrasonographically in patients with "idiopathic" pancreatitis and cholecystectomy offered in order to prevent further attacks.

Is this justified? In our opinion this recommendation is correct but probably not for the reason stated by the authors. This is more than just a case of academic hair-splitting as it again raises the whole area of the adequate investigation of gallstones in patients with an attack of acute pancreatitis. Is cholesterolosis a pathological link to acute pancreatitis *per se* or is it simply a marker of gallstone formation? There are a number of indicators from this paper which might lead one to reject the author's hypothesis.

Before moving onto this there is one perhaps not so small side issue. There was a discordant note struck when the authors gave their definition of acute pancreatitis: a compatible clinical picture along with a urinary amylase of > 1000 units/ml (normal range < 650 units/ml). This is less than twice the upper limit of normal. In our clinical practice, a diagnostic urinary amylase level is > 3,000 iu/l (normal serum range < 300 iu/l). Thus taking into account the different units, a urinary amylase is usually taken to be diagnostic if it is 10 × the upper normal serum level. As actual values were not given, one might be left wondering as to whether we would agree with a diagnosis of acute pancreatitis in all if not some of these cases.

Let us now return to the main issue. The overall incidence of acute pancreatitis (if we accept their definition) in this group of 434 patients with cholesterolosis was 28.8%. This is extremely high! Other series usually indicate an incidence of acute pancreatitis in patients with gallstones of up to 8% [9,10]. Yet this figure on reflection seems acceptable since patients with cholesterolosis are prone to form small gallstones and typically microliothiasis. Thus in the study from Bismuth's Unit, the incidence of acute pancreatitis was 22% in 68 patients with microliothiasis (diameter < 3mm) compared to 3.2% in 557 patients with larger stones [9]. Unfortunately, P. Parilla Paricio *et al.* gave no indication as to gallstone size in their study. Moreover in a retrospective study such as this, it is uncertain as to how thorough all the surgeons were in attempting to identify the presence of gallstones. Freund *et al.* showed that in 8% of their 63 patients with gallstone associated acute pancreatitis, small (1 – 2 mm) stones were only detectable after filtering the bile through gauze[11].

Of further note is that the incidence of acute pancreatitis was greatest in patients with cholesterolosis and no gallstones (49.1%) compared to those with cholesterolosis and gallstones (25.9%). If we accept the authors hypothesis that cholesterolosis is a separate aetiological factor to gallstones, it seems strange to find that the group with two aetiological factors has a smaller incidence of acute pancreatitis compared to the group with just one factor. It would have been valuable to know the overall incidence of acute pancreatitis amongst all patients with gallstones in their population.

A more consistent interpretation of these data might be that cholesterolosis is simply an indicator of a high propensity to form microlithiasis [12,13]. In some patients they might have all been passed during the attacks of acute pancreatitis and in others, they might have been "missed" at operation. Both the former [14,15] and the latter [11] are clearly documented.

It is of some interest that as long ago as 1957, Juniper and Burson noted duodenal bile crystals in 25% of patients with idiopathic pancreatitis [16]. These crystals, either of cholesterol or of calcium bilirubinaste are indicative of gallstone formation. This fact can be used to select out patients who in all probability have passed all their stones or have stones so small that they are not radiologically detectable [15,17,18].

The cholesterolosis polyp hypothesis is a variant of an old theme. Goldstein *et al.* in 1980 found 10 patients with idiopathic acute pancreatitis who had aggregates of bile pogments in duodenal aspirates. Eight of the patients agreed to cholecystectomy and suffered no further attacks [10]. Not surprisingly they proposed this as a mechanism for acute pancreatitis. These authors, however, did not report on a control group; if they had done they would have seen that such aggregates (known to surgeons as "sludge") are extremely common in a healthy population. A clear distinction between such "sludge" and true crystals must be made and requires the use of polarizing microscopy [15].

There is perhaps an alternative hypothesis linking cholesterolosis (irrespective of gallstones) to acute pancreatitis and that is the associated condition of cholesterolosis of the ampulla of Vater [20-22]. This would tie in with the notion of ampullary dyskinesia which has been shown by ERCP — manometric studies to be prevalent in patients with idiopathic pancreatitis [23,24]. Although much interest was shown in this form of "Odditis" by surgeons in the past, it has been difficult to document histologically in recent times despite the fact that it is well recognised [20,21]. Thus the association between gallbladder cholesterolosis, ampullary cholesterolosis and sphincter of Oddi dysfunction remains speculative and requires further investigation.

P. Parilla Paricio and colleagues are to be congratulated on highlighting again in such a definitive manner the important association between acute pancreatitis and cholesterolosis. Although in our opinion the true link is gallstones this in no way undermines the recommendation for cholestectomy in such cases.

The patient with acute pancreatitis and no immediate cause, and no stones on ultrasonography needs full investigation. This must include ERCP [13,24,25] and if need be duodenal bile crystal analysis [17,18] and repeat ("late") ultrasonography [15].

The detection of cholesterolosis, albeit not always radiologically easy [13] is a positive indication for cholecystectomy.

John P. Neoptolemos,
Reader in Surgery, University of Birmingham
Consultant Surgeon, Dudley Road Hospital
Brian Isgar,
Research Registrar, University Department of Surgery
Dudley Road Hospital, Birmingham, B18 7QH.

### REFERENCES

1. DeBolla, A.R. and Obeid, M.L. (1984) Mortality in acute pancreatitis. *Ann. Roy. Coll. Surg*, **66**, 184–186
2. Corfield, A.P., Cooper, M.J. and Williamson, R.C.N. (1985) Acute pancreatitis: A lethal disease of increasing incidence. *Gut;<ie.* **26**, 724–9
3. Goodman, A.J., Neoptolemos, J.P., Carr-Locke, D.L. Finlay, D.B.L. and Fossard, D.P. (1985) Detection of gallstones after acute pancreatitis. *Gut*; **26**, 125–32
4. Kune G. and Brough, W. (1989) Surgical intervention in sever acute pancreatitis: 476 cases in 20 years. *Ann. Roy. Coll. Surg.* **71**, 23–7
5. Ranson, J.H.C. (1982) Etiologic and prognostic factors in human acute pancreatitis: a review. *Ann. J. Gastoenterol.* **77**, 633–8
6. Corfield, A.P., Cooper, M.J., Williamson, R.C.N., Mayer, A.D., McMahon, M.J., Dickson, A.P., Shearer, M.G. and Imrie, C.W. (1985) Prediction of severity in acute pancreatitis: prospective comparison of three prognostic indices. *Lancet*, **ii**, 403–7
7. Thomson, H.J. (1985) Acute pancreatitis in north and north-east Scotland. *J. Roy. Coll. Surg.*, **30**, 104–11
8. MRC Multicenter Trial (1977) Death from acute pancreatitis. *Lancet*, **2**, 632–635
9. Houssin, D., Castain, G.D., Lemoine, J. and Bismuth, H. (1983) Microliothiasis of the gallbladder. *Surg. Gynecol. Obstet.* **157**, 20–4
10. Armstrong, C.P., Taylor, T.V., Jeacock, J. and Lucas, S. (1985) The biliary tract in patients with acute gallstone pancreatitis. *Br. J. Surg.* **72**, 551–5
11. Freund, H., Pfeffermann, R., Durst, A.L. and Rabinovici, N. (1976) Gallstone pancreatitis. Exploration of the biliary system in acute and recurrent pancreatitis. *Arch. Surg.* **111**, 1106–1107
12. Tilvis, R.S., Aro, J., Strandberg, T.E., Lempinen, M. and Miettinen, T.A. (1982) Lipid composition of bile and gallbladder mucosa in patients with acalculous cholesterolosis.. *Gastroenterology*, **82**, 607–15
13. Berk, R.N., Van der Vegt J.H. and Lichenstein, J.E. (1983) The hyperplastic cholecystoses: cholesterolosis and adenomyomatosis.. *Radiology*, **146**, 593–601
14. Mayer, A.D., McMahon, M.J. (1986) Gallstones and acute pancreatitis – is the association underestimated? (Abstract) *Br. J. Surg.* **71**, 905
15. Neoptolemos, J.P., Davidson, B.R., Winder, A.F. and Vallance, D. (1988) The role of duodenal bile crystal analysis in the investigation of "idiopathjic" pancreatitis. *Br. J. Surg.* **75**, 450–3
16. Juniper, K. and Burson E.N. (1957) Biloiary tract studies II. The significance of biliary crystals. *Gastroenterology*, **32**, 175–209
17. Block, M.A. and Priest, R.J. (1967) Acute pancreatitis related to grossly minute stores in a radiographically normal gallbladder. *Ann. J. Dig. Dis.*, **12**, 934–8
18. Negro, P., Flati, G., Flati, D., Arrowska, B., Tuscano, D. and Carboni, M. (1984) Occult gallbladder microlithiasis causing acute recurrent pancreatitis. *Acta. Clin. Scand.*, **150**, 503–6
19. Goldstein, F., Kucer, F.T., Thornton, J.J. and Abramson, J. (1980) Acute and relapsing pancreatitis caused by bile pigment aggregates and diagnosed by biliary drainage. *Ann. J. Gastroenterol.*, **74**, 225–30
20. Nardi, G.L. (1973) Papillitis and stenosis of the sphincter of Oddi. *Surg. Clin. N. Amer.*, **53**, 1149–60
21. Moody, E.G., Becker, J.M. and Potts, J.R. (1983) Transduodenal sphincteroplasty and transampullary septectomy for postcholecystectomic pain. *Ann. Surg..*, **197**, 627–34
22. Neoptolemos, J.P., Bailey, I.S. and Carr-Locke, D.L. (1988) Sphincter of Oddi dysfunction: results of treatment by endoscopic sphincterotomy. *Br. J. Surg.*, **75**, 454–9
23. Toouli, J., Roberts-Thomson, I.C., Dent, J. and Lee, J. (1985) Sphincter of Oddi motility disorders in patients with idiopathic recurrent pancreatitis. *Br. J. Surg.*, **72**, 859–63
24. Venu, R.P., Geennan, J.E., Hogan, W., Stone, J., Johnson, G.K. and Soergel, K. (1989) Idiopathic recurrent pancreatitis. An approach to daignosis and treatment. *Dig. Dis. Sci.*, **34**, 56–60
25. Lee, M.J.R., Lai, E.C.S. and Wong, J. (1986) Endoscopic retrograde cholangiopancreatography after acute pancreatitis. *Surg. Gynaecol. Obstet.*, **163**, 354–8

